# Cisplatin treatment induced interleukin 6 and 8 production alters lung adenocarcinoma cell migration in an oncogenic mutation dependent manner

**DOI:** 10.1186/s12931-020-01389-x

**Published:** 2020-05-20

**Authors:** Edit Kiss, El Husseiny Mohamed Mahmud Abdelwahab, Anita Steib, Emoke Papp, Zsofia Torok, Laszlo Jakab, Gabor Smuk, Veronika Sarosi, Judit Erzsebet Pongracz

**Affiliations:** 1 Department of Pharmaceutical Biotechnology, Faculty of Pharmacy, University of Pecs, 2 Rokus Str, Pecs, Hungary; 2 Humeltis Ltd, 20 Ifjusag Str, Pecs, Hungary; 3 Szentagothai Research Centre, University of Pecs, 20 Ifjusag Str, Pecs, Hungary; 4 Department of Internal Medicine, Medical School and Clinical Centre, University of Pecs, 13 Ifjusag Str, Pecs, Hungary; 5 Department of Surgery, Medical School and Clinical Centre, University of Pecs, 13 Ifjusag Str, Pecs, Hungary; 6 Department of Pathology, Medical School and Clinical Centre, University of Pecs, 13 Ifjusag Str, Pecs, Hungary

**Keywords:** Non-small cell lung cancer, Adenocarcinoma, EGFR, Cisplatin, Erlotinib, IL-6, IL-8

## Abstract

**Background:**

The predominant metastatic site of lung cancer (LC) is the brain. Although outdated, conventional cisplatin treatment is still the main therapeutic approach for patients with advanced non-small cell lung cancer (NSCLC), since targeted therapy that offers better tumor control is not always possible. In the present study brain metastasis associated cytokine expression was investigated in primary NSCLC adenocarcinoma (AC) tissues with known oncogenic mutations in the presence or absence of platina based and tyrosine kinase inhibitor (TKI) drugs.

**Methods:**

Primary lung tumor samples were isolated, DNA was sequenced and then the samples were grouped based on mutation. Experiments were also performed using KRAS mutant A549 and EGFR mutant PC-9 cells. Drug response was analyzed in three dimensional (3D) tissue cultures. We assessed drug response and IL-6 and IL-8 cytokine expression in relation to cellular invasion using ATP dependent cell viability, qRT-PCR analysis, cytokine bead array, and migration assay.

**Results:**

In 3D co-cultures, primary NSCLC derived cells harboring EGFR mutation responded better to erlotinib treatment than KRAS mutant or KRAS/EGFR wild type (WT) cancer cells. In contrast, under the same culture conditions KRAS/EGFR WT or KRAS mutant cancer cells are more sensitive to cisplatin than EGFR mutant cells. Drug response and pro-inflammatory cytokine production varied depending on the driver mutations. Cisplatin but not erlotinib increased both IL-6 and IL-8 secretion and only IL-6 increased cellular migration and proliferation.

**Conclusion:**

In vitro assays are available to determine the response to planned therapeutic approach of lung cancer subtypes. The sequence of administration of therapeutic drugs determines cytokine production and therefore therapeutic response.

## Background

Effective therapy of lung cancer (LC) is still one of the greatest challenges in cancer care. Despite the great promises of novel immunotherapies [1] the vast majority of newly diagnosed LC cases are treated with conventional chemotherapy as the cancer is already metastasized by the time of diagnosis [2]. In such a fast progressing disease, the slower acting immunotherapies can only offer treatment advantage in specific cases and mainly in younger patients.

The vast majority (around 80%) of LC-s belong to the non-small cell lung cancer (NSCLC) type where the largest subtype is adenocarcinoma (AC) [3, 4]. To find the best therapeutic approach, key mutations including epidermal growth factor receptor (EGFR), Kirsten rat sarcoma viral oncogene homolog (KRAS), echinoderm microtubule-associated protein-like 4–anaplastic lymphoma kinase (EML4–ALK), and recently B-Raf proto-oncogene serine/threonine kinase (BRAF) are routinely tested [5–7]. Although radical improvements have not been observed in survival statistics, targeted therapies can be used to slow down progression in the presence of certain mutations. In case of EGFR mutation, erlotinib, gefitinib, and the second generation afatinib are recommended [3, 4].

The predominant metastatic site of both NSCLC and SCLC is the brain, and up to 68% of patients with mediastinal lymph node metastasis eventually have brain metastasis [8]. Even in comparison with other common epithelial malignancies, the frequency of brain metastasis is the highest in LC-s [9]. Brain metastasis is significantly higher in patients with confirmed EGFR mutations compared to those with wild type EGFR [10]. EGFR mutation with exon 19 deletion induces multiple small brain metastasis with smaller peri-tumoral brain edema than occurs in those without EGFR mutations [11]. The EGFR tyrosine kinase inhibitors (TKI) gefitinib and erlotinib have been tested in patients with NSCLC brain metastasis [12]. Similarly to primary tumors, the response of brain metastasis to EGFR inhibitors is better in patients with activating EGFR mutations while the activity of these drugs in individuals with wild-type EGFR metastatic disease is modest at best [12].

The response to erlotinib and gefitinib in chemotherapy-naïve, non-smoker patients with brain metastases was significantly better than smoker patients with brain metastasis, indicating that there may be additional mutations that are the result of cigarette smoking or chemotherapy that facilitate brain metastasis. Ineffectiveness of targeted therapy is more often the case in patients who received platinum based (carboplatin or cisplatin) chemotherapy prior to targeted therapy. As both carboplatin, and especially cisplatin markedly increases the mutation rate [7], added mutations could alter the response to therapy. Additionally, the molecular microenvironment changes upon therapy and can affect the therapeutic outcome; increased soluble chemokines and cytokines including interleukin 8 (IL-8) and interleukin 6 (IL-6) have been detected in a variety of cancers and such cytokine surges can promote metastasis [1, 3].

In the current work our primary aim was to investigate whether the treatment response of EGFR mutant tumors could be mimicked in vitro and used as a in drug selection studies [13]. We also aimed to study whether IL-8 and IL-6 cytokine production is triggered upon therapy which could ultimately affect cellular proliferation and migration. This may ultimately be of use in the selection of the best available treatment for these cancers.

## Materials and methods

### Cell cultures

KRAS-mutant A549 (p.*G12S* c.34G > A) human lung adenocarcinoma cell line (American Type Cell Culture Collection, Rockville, MD, USA) was grown in DMEM (Lonza, Walkersville, MD, USA) supplemented with 10% FBS (Biowest, Nuaillé, France), 1% L-glutamine (Lonza, Walkersville, MD, USA), 2% penicillin/streptomycin (Hyclone, Logan*,* UT*,* USA*),* 1% HEPES (Lonza, Walkersville, MD, USA), 1% non-essential amino-acids (Lonza, Walkersville, MD, USA), 1% PBS/beta-mercaptoethanol). EGFR-mutant PC-9 (exon19del E746–A750) human lung adenocarcinoma cell line (Sigma-Aldrich, St. Louis, Missouri, USA) was maintained in RPMI 1640 (Corning, NY, USA) containing 10% FBS, 1% L-glutamine and 2% penicillin/streptomycin at 37 °C in humidified atmosphere containing 5% CO_2_. Primary human lung fibroblasts (NHLF) were cultured in FGM-2 according to the manufacturers’ recommendations (Lonza, Walkersville, MD, USA).

### Primary lung cancer tissues

Lung tissue samples were collected during tumor resections at the Department of Surgery, University of Pecs, Hungary. Pleural effusion samples were collected at the Division of Pulmonology, Department of Internal Medicine, Clinical Centre, the University of Pecs, Hungary. The project was approved by the Ethical Committee of the University of Pecs (2014-RIKEB-5329-EKK) and the Medical Research Council of Hungary (366/2015 (46945–1/2015/EKU)). Patients had given written informed consent and their samples were independently coded and treated anonymously. Sequencing of the samples was part of the routine pathology testing. Patient data is summarized in Table 1.

**Table 1:** Patient list

No	Mutation	Histology	T	N	M
1	EFGR/KRAS WT	Adenocc	T2	N1	Mx
2	EFGR/KRAS WT	Adenocc	T2	N1	M1
3	EFGR/KRAS WT	Adenocc	T1	N1	Mx
4	KRAS MUTANT	Adenocc	T2	N1	Mx
5	KRAS MUTANT	Adenocc	T2	N0	Mx
6	KRAS MUTANT	Adenocc	T2b	N0	Mx
7	KRAS MUTANT	Adenocc	T3	N2	Mx
8	KRAS MUTANT	Adenocc	T2	N0	Mx
9	KRAS MUTANT	Adenocc	T1	N2	Mx
10	KRAS MUTANT	Adenocc	T2	N2	Mx
11	KRAS MUTANT	Adenocc	T1	N1b	Mx
12	EGFR MUTANT	Adenocc	T2b	N1	Mx
13	EGFR MUTANT	Adenocc	T3	Nx	M1
14	EGFR MUTANT	Adenocc	T1	N1	Mx
15	EGFR MUTANT	Adenocc	T2	N3	M1

### Primary tumor cell isolation

Solid tumor tissues were resected, and viable tumor areas were selected by a certified lung pathologist. Tissue samples were placed into sterile MACS® Tissue Storage Solution (Miltenyi Biotec, Auburn, USA), sliced then digested using a gentleMACS Dissociator (Miltenyi Biotec, Auburn, USA) according to the manufacturer’s recommendation (Miltenyi Biotec, Auburn, USA). Briefly, solid tumor tissues were digested (40 min, at 37 °C) in RPMI 1640 supplemented with an enzyme mix provided by the manufacturer. Cells were pelleted, resuspended in RPMI 1640, passed through a cell strainer, and then centrifuged. The pellet was resuspended in DMEM. Cells were cryo-preserved using Cryo-SFM according to the manufacturer’s recommendation (PromoCell, Heidelberg, Germany) and stored at -80 °C until used.

### In vitro three dimensional (3D) lung aggregates

NHLF and A549 or PC9 were mixed in 1:1 ratio and a total of 30,000 cells/well were pipetted onto a low-attachment 96-well U-bottom plate (Corning, NY, USA). Cells were sedimented (600 g for 10 min) and cultured at 37 °C and 5% CO_2_ in mixed DMEM:FGM-2 or RPMI:FGM-2 media at 1:1 ratio, respectively [14].

### Drugs and reagents

Cisplatin (Accord Healthcare) was purchased from the University Pharmacy, University of Pecs, Hungary). Erlotinib was purchased from Selleckem (Houston, TX, USA). Drugs were added to cells at final concentration of 30 nM cisplatin, and various concentrations (1 nM, 10 nM, 100 nM and 1 μM) of erlotinib for 48 h. The choice of erlotinib optimal concentration was determined using a cell viability assay. Recombinant human IL-6 and IL-8 was purchased from R&D Systems (Minneapolis, MN, USA) and used at a final concentration of 100 ng/ml for 48 h.

### Cell viability assay

CellTiter-Glo Luminescent Cell Viability Assay Kit (Promega Corp., Madison, WI, USA) was used to evaluate cytotoxicity after drug treatment. Co-cultures were seeded into 96-well plates, after 24 h incubation 2D or 3D cell cultures were treated with erlotinib and/or cisplatin. After incubation for 48 h at 37 °C, 100 μl of CellTiter-Glo reagent were added and luminescence measured with EnSpire® Multimode Plate Reader (PerkinElmer, Waltham, Massachusetts, USA). Each experiment was performed in triplicates for each concentration and repeated three times (*n* = 3).

### RNA isolation, cDNA synthesis

Total RNA was extracted using NucleoSpin RNA II isolation kit according to manufacturer’s protocol (Macherey-Nagel, Düren Germany). RNA concentration was measured by Nanodrop (ThermoFisher Scientific, Waltham, Massachusetts, USA). One microgram of total RNA was used to generate cDNA using High-Capacity cDNA Reverse Transcription Kit (ThermoFisher Scientific, Waltham, Massachusetts, USA).

### Quantitative (q)RT-PCR

qRT-PCR-s were carried out using the SensiFAST™ SYBR® Hi-ROX Kit (BioLine, London, UK). Amplifications were done on a StepOnePlus system (Applied Biosystems, Foster City, CA, USA). Gene expression was analysed with StepOne software, using the housekeeping gene ß-actin as reference standard. The primer sequences are listed in Table 2. The cycling parameters were the following: one cycle 95 °C for 2 min, 40 cycles at 95 °C for 5 s and 60 °C for 30 s. The relative quantities (RQ) were calculated using the 2^-ddCt^ method.

**Table 2:** PCR primer sequences

Target gene	Forward primer	Reverse primer
**human β-actin**	GCGCGGCTACAGCTTCA	CTTAATGTCACGCACGATTTCC
**human IL-6**	AGGGCTCTTCGGCAAATGTA	GAAGGAATGCCCATTAACAACAA
**human IL-8**	CAGTTTTGCCAAGGAGTGCTA	AACTTCTCCACAACCCTCTGC

### Cytokine production

Inflammatory cytokine protein levels were quantified after cisplatin and/or erlotinib treatment using BD™ CBA Human IL-6 and IL-8 Flex Set Assays CBA (BD Biosciences, San Diego, CA, USA) according to the manufacturer’s instructions. Cytometric Bead Arrays (CBA) were then run on BD FACSCanto II flow cytometer (BD Immunocytometry Systems, Erembodegen, Belgium) and analyzed.

### 3D wound healing bioassay

A549 and PC-9 cells were cultured on T-25 flasks until they reached 80% confluence, then treated with 200 μL NanoShuttle-PL overnight at 37 °C, 5% CO_2._ After 24 h incubation single cell suspensions were made and cells were seeded to the 6-well repellent plate at a density of 1.2 × 10^6^ cells/well. A 6-well magnet was placed on the top of the plate for 5 h to levitate the cells and induce ECM formation [13, 15]. After incubation cells were collected and added to 24-well repellent plate at a concentration of 2 × 10^5^ cells/well. A 24-well ring magnet was placed below the plate for 15 min to allow cells to aggregate into the magnet’s ring shape. Then, the cells were exposed to cisplatin (30 nM) or erlotinib (100 nM). Cell growth was documented by taking pictures at every 6 h for 24 h using an EVOS® FL Imaging System.

### Scratch assay

Cells were grown to 90% confluence in 24 well plates (Corning Costar, Darmstadt, Germany) and wound was created in each culture by scratching the cellular monolayers. Fresh medium supplemented with cisplatin (30 nM) or erlotinib (100 nM) in the presence or absence of 100 ng/ml IL-6 or IL-8 was added to the cell cultures, respectively. Wound healing was monitored by the decrease of gap area taking images with EVOS light microscopy (Thermo Fisher Scientific, Waltham, USA) at regular intervals and the gap area was quantified using ImageJ software.

### Statistical analysis

Data are presented as mean ± standard error of mean (SEM), and statistical analysis was performed using one-way ANOVA test. *p* < 0.05 was considered as significant.

## Results

### Drug sensitivity of primary human lung adenocarcinomas can be predicted in in vitro tissue cultures

**Fig. 1:**
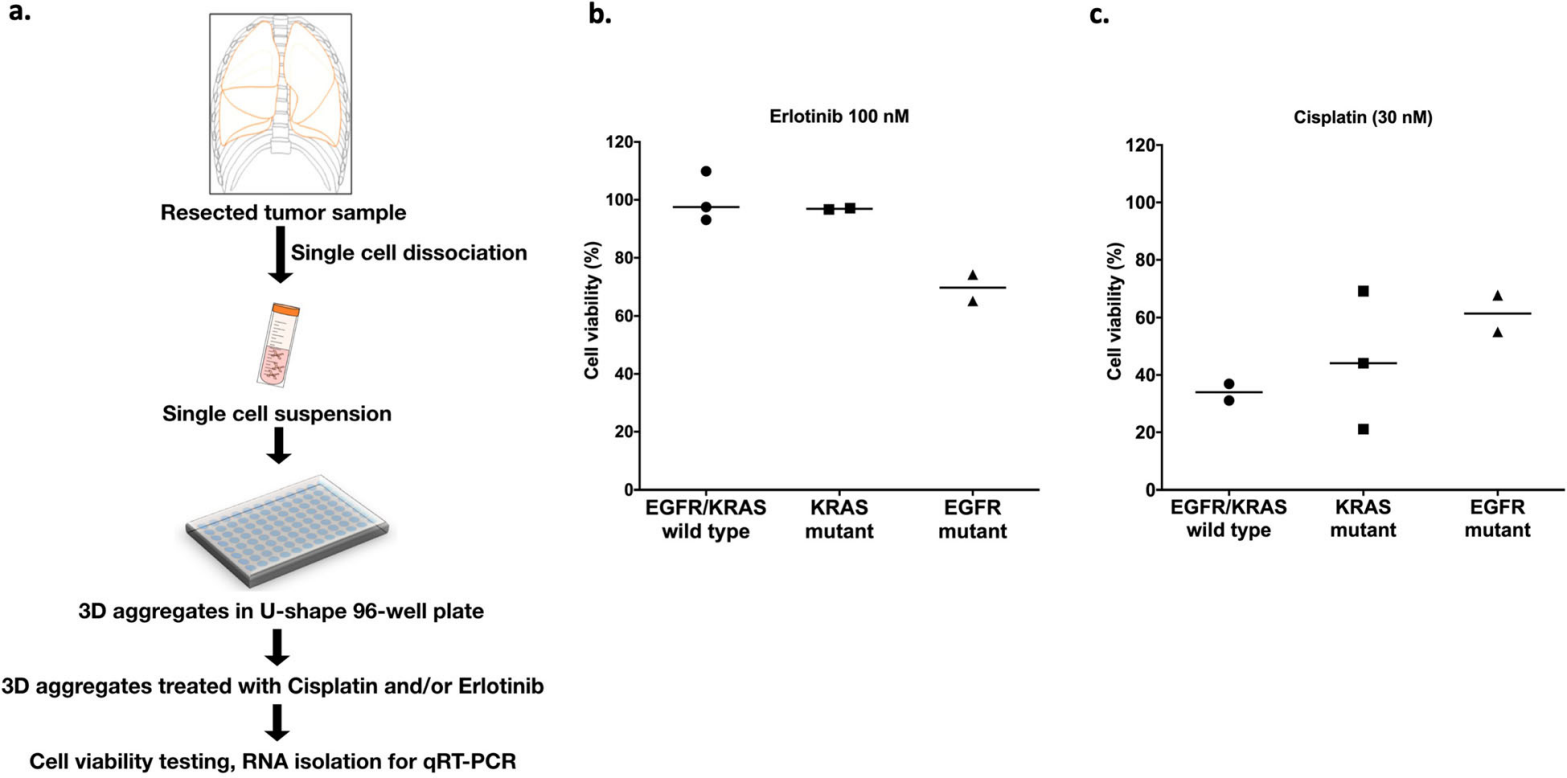
Primary sample processing protocol and 3D cell viability assay following erlotinib and cisplatin treatment in vitro. **a** Summary of patient samples processing protocol. **b** Percentage of cell viability following erlotinib (100 nM) treatment for 48 h compared to untreated samples (WT *n* = 3, KRAS *n* = 2 and EGFR *n* = 2). Data are presented as scatter plot of individual points with mean. **c** Percentage of cell viability following cisplatin (30 nM) treatment for 48 h (WT *n* = 2, KRAS *n* = 3 and EGFR *n* = 2)

Fifteen patient samples were used in the study, all of whom had primary lung adenocarcinoma (AC). The samples from 8 patients had KRAS mutations, 4 had activating EGFR mutation and 3 patients had wild type (WT) EGFR and KRAS genes. Patient information is summarized in Table 1. All the samples were freshly cryopreserved as single cell suspensions after surgery, and then thawed for testing when the mutation analysis became available. The protocol is summarized in Fig. 1a. Not all of the samples provided enough material to be used in every experiment, hence there are differences in the number of freshly isolated samples in individual experimental settings.

To investigate the drug response of primary lung AC-s with various mutation background in vitro, 3D aggregate cultures were formed from primary cancer cells, after which the cultures were exposed to cisplatin (30 nM) or erlotinib (100 nM). Studies indicate that in vitro drug sensitivity assays replicate the clinically proven drug response [16–18]. Patients with exon 19 deletions or exon 21 substitution (L858R) in the EGFR gene were responsive to TKI (erlotinib), while samples with KRAS mutation or WT KRAS/EGFR genes were not sensitive to erlotinib (Fig. 1b) [19–21]. Additionally, the in vitro drug sensitivity assay confirmed that cisplatin was more cytotoxic to cells with WT KRAS/EGFR mutations than with KRAS mutations and was least effective in those with EGFR mutations (Fig. 1c) [22].

### Cisplatin and erlotinib treatment alters cell viability and migration

**Fig. 2:**
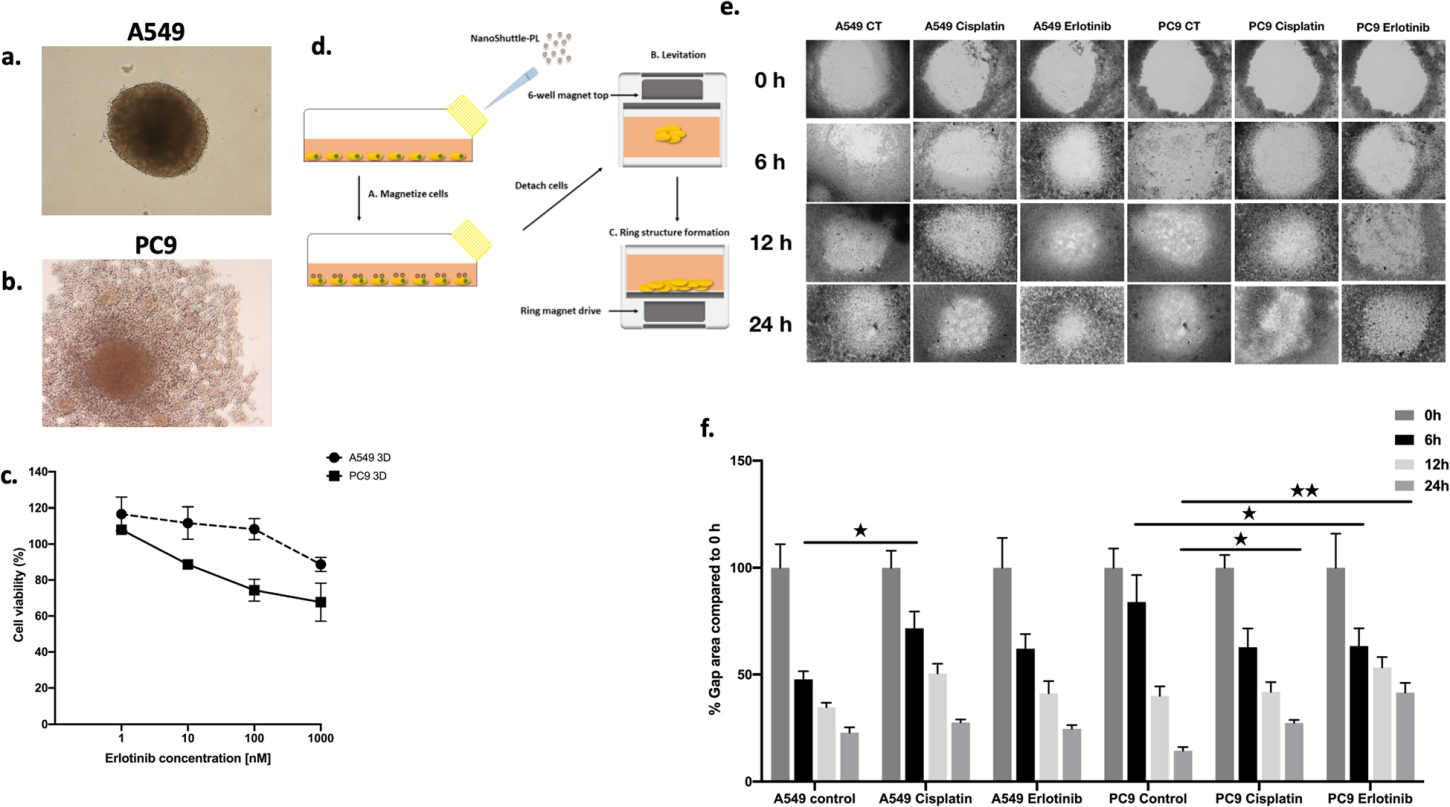
A549 and PC-9 cell viability and migration alteration post cisplatin and erlotinib treatment. **a** 200 μm light microscopy images of 3D lung model of NHLF-A549 (75–25%) co-culture **b** 200 μm light microscopy images of 3D lung model of NHLF-PC-9 (75–25%) co-culture. **c** Percentage of cell viability expectancy for A549 and PC-9 versus erlotinib concentrations (1, 10, 100, 1000 nM). Data are presented as individual survival points ± SEM. **d** Summary of the NanoShuttle magnetic beads migration assay protocol. **e** A549 and PC-9 migration capacity at different time points (0, 6, 12, 24 h) following cisplatin (30 nM) and erlotinib (100 nM) treatment. **f** Quantification of migration capacity using gap area measurement. Data are presented as % of Gap area compared to 0 h ± SEM and significant changes are marked as ★ (*P* < 0.05) and ★★ (*P* < 0.001)

To be able to investigate the microenvironment in more detail, two human lung AC cell lines with different EGFR status were treated with erlotinib in 3D co-cultures (Fig. 2a, b and c) [23]. Similarly to primary lung AC cells, cell viability of KRAS mutant A549 aggregate co-cultures were barely affected by erlotinib at lower concentrations (1–100 nM) and some decrease in viability was only detected at 1000 nM, far above the sensitivity of the EGFR mutant (exon 19 deletion) PC-9 cell cultures that reacted to erlotinib at 10 nM (Fig. 2c).

To test whether cell proliferation and migration is affected by the two above drugs, a novel cellular migration test was performed. In the magnetic migration test the KRAS mutant A549 and the EGFR mutant PC-9 lung adenocarcinoma cell lines were used in 3D mono-cultures (Fig. 2d). 30 nM cisplatin treatment of the KRAS mutant A549 cells only transiently delayed gap-closure and by 24 h the gap was closed to the same level as in the untreated control (Fig. 2e and f). Erlotinib had no significant effect (Fig. 2e and f) in the KRAS mutant cultures. The EGFR mutant PC-9 cell line responded well to erlotinib and a significantly larger gap area was preserved even at 24 h of incubation compared to untreated and even to cisplatin treated control (Fig. 2e, f).

### Cisplatin significantly increases pro-inflammatory cytokine production

**Fig. 3:**
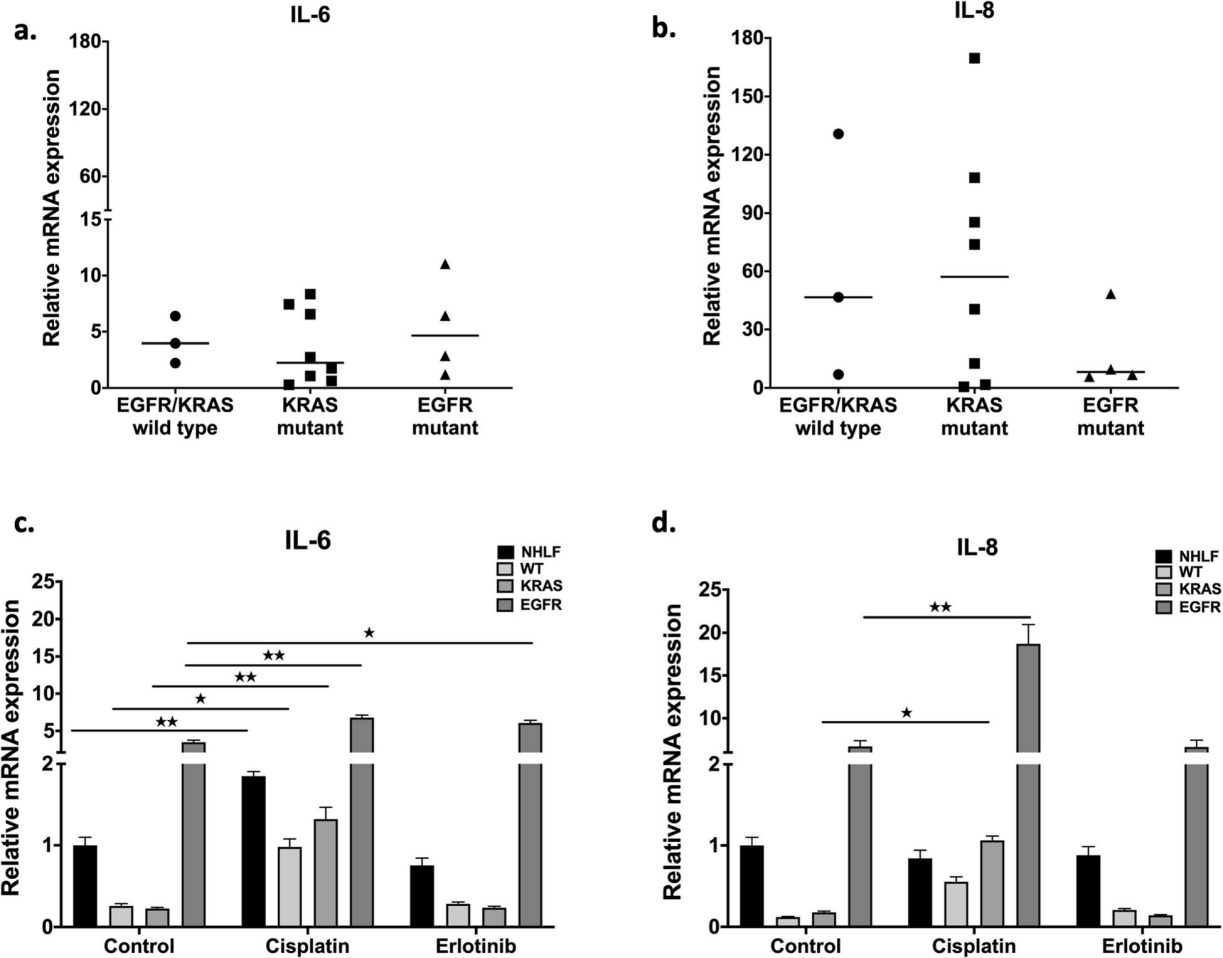
IL-6 and IL-8 production in primary lung AC tissues. **a** IL-6 mRNA expression level in untreated primary samples (WT *n* = 3, KRAS *n* = 8 and EGFR *n* = 4). Data are presented as scatter plot of individual points with mean. **b** IL-8 mRNA expression level in untreated primary samples (WT *n* = 3, KRAS *n* = 8 and EGFR *n* = 4). Data are presented as scatter plot of individual points with mean. **c** IL-6 mRNA expression levels of selected primary samples following cisplatin and erlotinib treatment. Data are presented as relative mRNA expression ± error and significant changes were tested with one sample t-test and marked as ★ (*P* < 0.05) and ★★ (*P* < 0.001). **d** IL-8 mRNA expression levels of selected primary samples following cisplatin and erlotinib treatment. Data are presented relative mRNA expression ± error and significant changes were tested with one sample t-test and marked as ★ (*P* < 0.05) and ★★ (*P* < 0.001)

To test whether primary, patient derived tumor samples express pro-inflammatory cytokines that influence cellular proliferation and migration, mRNA levels of IL-6 and IL-8 were measured. The levels of both IL-6 and IL-8 varied greatly amongst individual patient samples (Fig. 3a, b). The only notable tendency was that while in the EGFR mutant primary AC samples IL-6 levels were higher than in KRAS or WT samples, IL-8 levels were the lowest. To test whether cisplatin and erlotinib affect IL-6 and IL-8 cytokine expression, WT, KRAS and EGRF mutant tumor samples were treated with cisplatin (30 nM) or erlotinib (100 nM), and then cytokine mRNA levels were measured (Fig. 3c, d). Cisplatin treatment increased both IL-6 and IL-8 message levels in all sample types, especially in the EGFR mutant tumor samples (Fig. 3c, d). In contrast, erlotinib did not influence cytokine production in WT or KRAS mutant primary samples, but did increase IL-6 levels in samples with EGFR mutation (Fig. 3c, d).

**Fig. 4:**
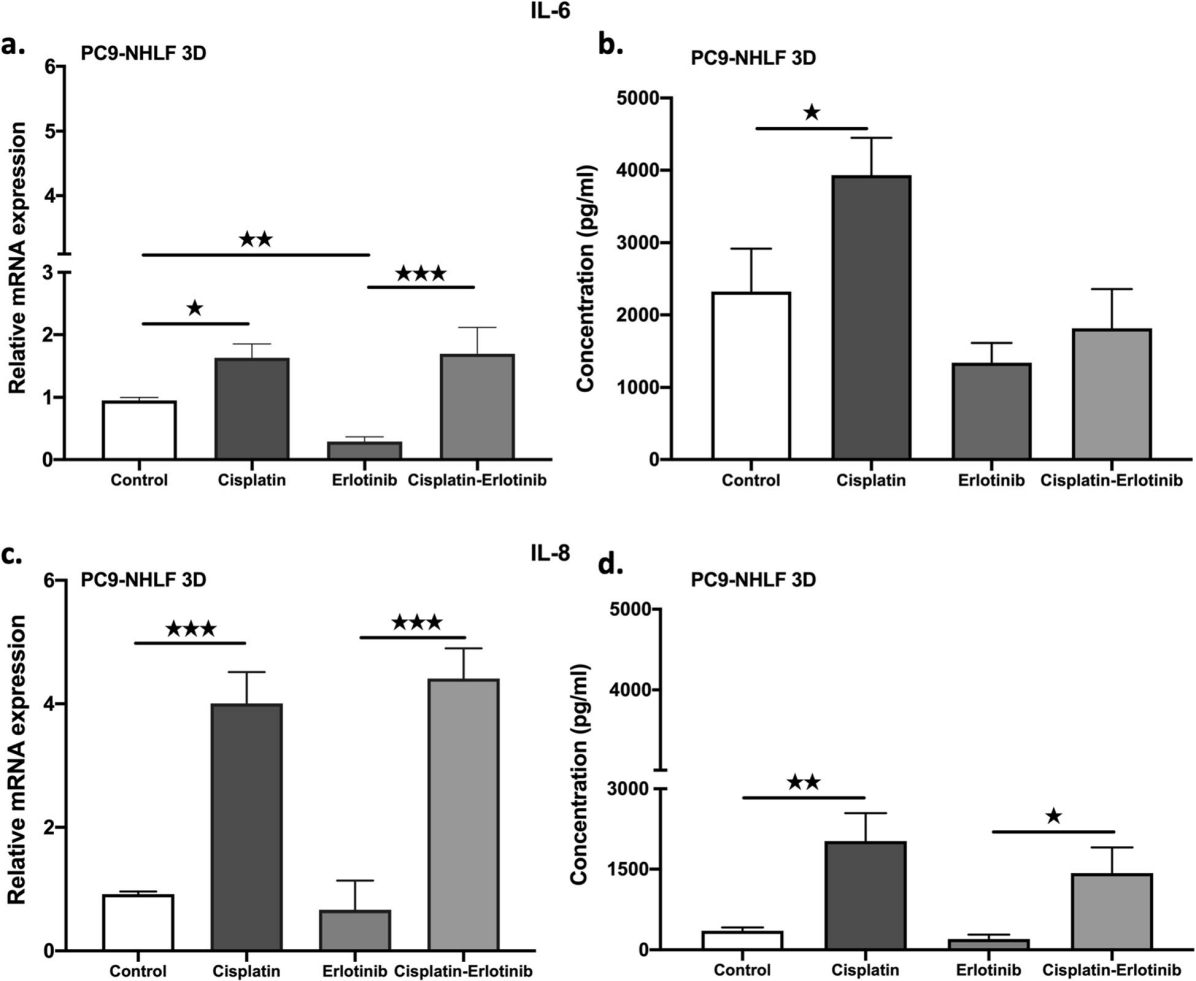
IL-6 and IL-8 cytokine production following cisplatin (30 nM) and erlotinib (100 nM) treatment of PC-9-NHLF lung aggregates. **a** relative IL-6 mRNA expression following cisplatin treatment. **b** relative IL-6 protein production following treatment **c** relative IL-8 mRNA expression following treatment **d** relative IL-8 protein production following treatment. Data are presented as relative mRNA expression ± SEM, protein concentration (pg/ml) ± SEM and significant changes are marked as ★ (*P* < 0.05), ★★ (*P* < 0.001) and ★★★ (*P* < 0.0002)

However, there was a great deal of variation between different in primary samples, with and the greatest differences observed between samples with of EGFR mutations. Therefore experiments were performed using the EGFR mutant AC cell line, PC-9. Pro-inflammatory IL-6 and IL-8 cytokine production in EGFR mutant PC-9 cell lines in 3D cultures were measured after cisplatin or erlotinib treatment. It was also tested whether cisplatin pre-treatment followed by erlotinib treatment affected cytokine production. Both inflammatory cytokine IL-6 and IL-8 production was significantly increased at the mRNA level after cisplatin mono-treatment or cisplatin and erlotinib combination treatment, while erlotinib alone did not increase message levels for either cytokine (Fig. 4a-d). Protein levels of both IL-6 and IL-8 were also tested, but elevated protein levels were only detected when the cell cultures were exposed to cisplatin. In fact, erlotinib reduced cisplatin induced IL-6 protein expression (Fig. 4b).

**Fig. 5:**
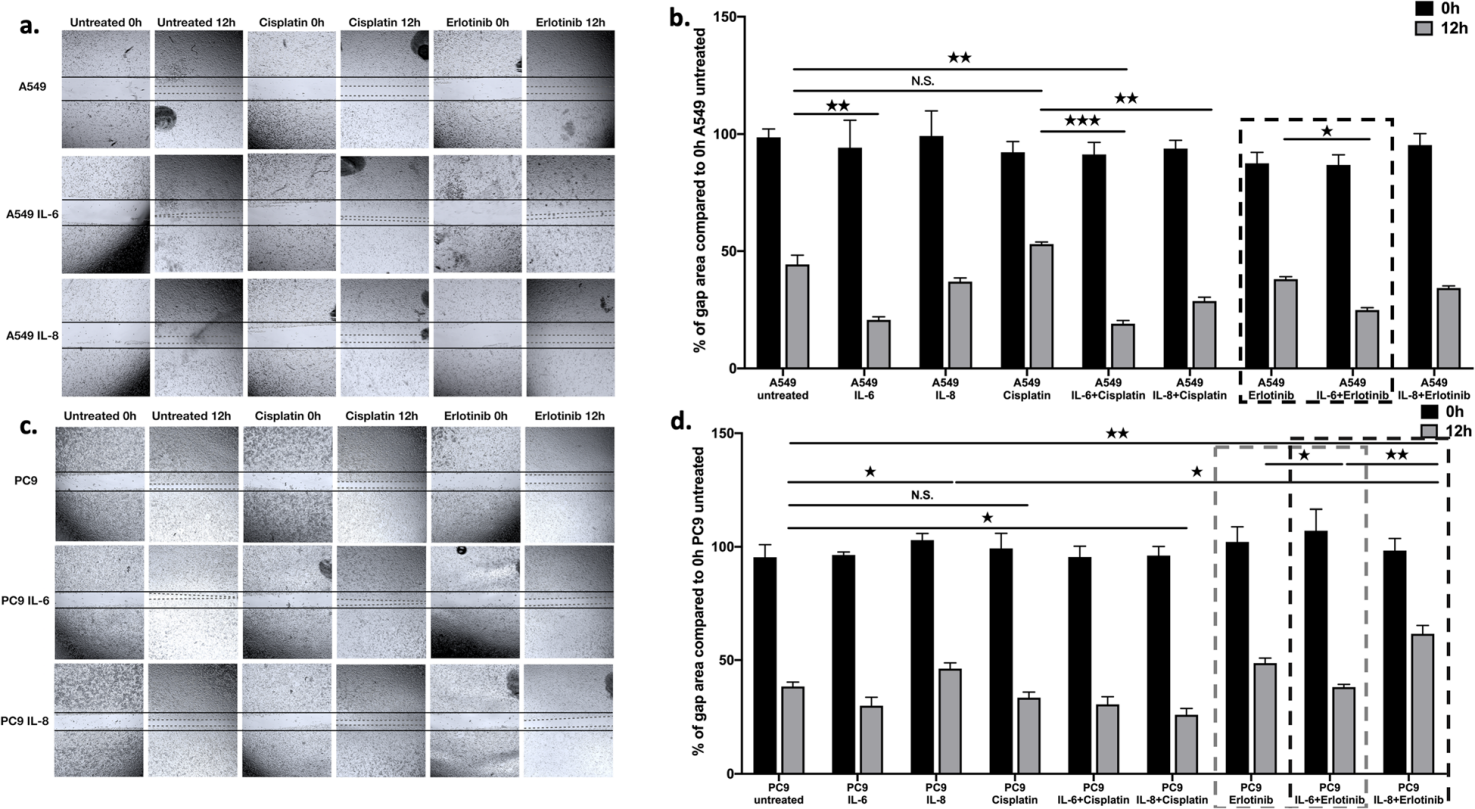
Scratch assay of KRAS (A549) and EGFR (PC-9) mutant cell lines following cisplatin, erlotinib, IL-6 and IL-8 mono and combination treatment. **a** Changes in gap area over time (12 h) and treatment of KRAS mutant cell line (A549) using IL-6 (100 ng/ml), IL-8 (100 ng/ml) and cisplatin (30 nM) or erlotinib (100 nM). Magnification is 10x. This is a single experiment representative of *n* = 3. **b** Quantification of gap area in KRAS mutant cell line (A549) cultures. Data are presented as % of gap area compared to gap area of untreated A549 cell layers SEM and significant changes are marked as ★ (*P* < 0.05), ★★ (*P* < 0.001) and ★★★ (*P* < 0.0002). **c** Changes in gap area over time (12 h) and treatment of EGFR mutant cell line (PC-9) using IL-6 (100 ng/ml), IL-8 (100 ng/ml) and cisplatin (30 nM) or erlotinib (100 nM). Magnification is 10x. Representative picture of *n* = 3. **d** Quantification of gap area in EGFR mutant cell line (PC-9) cultures. Data are presented as % of gap area compared to gap area of untreated PC-9 cell layers SEM and significant changes are marked as ★ (*P* < 0.05), ★★ (*P* < 0.001) and ★★★ (*P* < 0.0002)

To test whether the presence of cytokines can modulate cellular migration and proliferation, scratch assays were performed in the presence or absence of the two drugs and/or cytokines. The addition of IL-6 (100 ng/ml) significantly reduced the gap area by inducing cellular proliferation and migration in the KRAS mutant A549 cultures (Fig. 5a, b). This was also the case if the cultures were treated with cisplatin or erlotinib in the presence of IL-6. IL-8 had no remarkable effect on gap closure in the KRAS mutant cell lines (Fig. 5a, b). In contrast, adding IL-6 had no significant effect on EGFR mutant PC-9 cells. PC-9 cultures responded to IL-6 during erlotinib treatment when added IL-6 promoted gap closure. More interestingly, IL-8 inhibited gap closure in the PC-9 cell line and even more so in the presence of erlotinib (Fig. 5c, d). Cisplatin was unable to slow down gap closure in the PC-9 cell cultures (Fig. 5c, d) and in the presence of cisplatin, added IL-6 and IL-8 accelerated the process (Fig. 5c, d).

## Discussion

Inflammation-associated cancer progression has become widely acknowledged in the past decades [24]*.* While IL-6 and IL-8 both promote angiogenesis, tumor cell survival, chemoresistance, and migration [25, 26]; it was the high IL-6 serum levels which was associated with poor survival rate in advanced NSCLC. This is due to increased drug resistance and reduced drug-induced apoptosis [27–30].

One of the widely used chemotherapeutic drugs in treatment of advanced cancers is cisplatin, which triggers inflammatory cytokine IL-6 and IL-8 production [17]. Cisplatin, apart from being strongly mutagenic [31] induces upregulation of both IL-6 and IL-8 via activation of the NFκB signaling pathway [18]. Moreover, elevated levels of pro-inflammatory cytokines can increase chemoresistance [29]. Elevated levels of IL-6 is also associated with increased permeability of the blood brain barrier (BBB) [32].

In clinical trials, platinum-based chemotherapy combined with EGFR-TKI had no survival benefits in advanced NSCLC [33–36], although preclinical studies indicated otherwise [37]. Using our methodology, we were able to preserve primary LC tissues and generate 3D aggregate cultures for in vitro drug sensitivity testing when sequencing data became available. The methodology allowed us to demonstrate that in vivo patient data and in vitro drug sensitivity tests provide highly similar results. We have shown that primary tumors with activating EGFR mutation were the least responsive to cisplatin while tyrosine kinase inhibition was only effective in the presence of activating EGFR mutation. Additionally, the level of IL-6 was the highest in the patient group with activating EGFR mutation. If patients were to be pre-treated with cisplatin, IL-6 levels can increase even further. As IL-6 negatively affects the BBB, increased brain metastasis can be further expected from the activating mutant EGFR AC-s if treated with cisplatin. It has also been demonstrated that erlotinib doesn’t increase IL-6 but high IL-6 levels can reduce the beneficial effects of TKI. In contrast, the presence of IL-8 did not reduce the tumor cell proliferation effect of erlotinib. It was also shown that erlotinib can inhibit cisplatin induced IL-6 secretion and accelerate cellular migration.

## Conclusions

Drug response can be effectively tested on primary cancer tissues in vitro [6, 13, 15, 21].

Somatic mutations of EGFR and KRAS are characteristic mutations in lung AC-s that promote accelerated tumor growth [38] and also affect drug response [39]. Preceding clinical therapy with an in vitro drug sensitivity test on a small number of tumor cells, could allow even individual cytokine responses to be detected, indicating clinical response to treatment. After further clinical validation of the above methods using a larger sample pool, such technique could become a valuable tool assisting the prediction of treatment response.

In cancer therapy the best treatment depends on the available drug, the sequence of administration, the patients’ general conditions and co-morbidities that alter the tumor microenvironments and hence their drug responses [40]*.* Based on our study, there is a possibility to test individual drug response using a great variety of output readings which all together provides additional information for predicting individual therapy response.

## Data Availability

All data generated and analyzed during the current study are available from the corresponding author on reasonable request.

## Abbreviations

LC: Lung cancer
NSCLC: Non-small cell lung cancer
AC: Adenocarcinoma
TKI: Tyrosine kinase inhibitor
3D: Three dimensional
IL: Interleukin
EGFR: Epidermal growth factor receptor
KRAS: Kirsten rat sarcoma viral oncogene homolog
EML4–ALK: echinoderm microtubule-associated protein-like 4–anaplastic lymphoma kinase
BRAF: B-Raf proto-oncogene serine/threonine kinase
WT: Wild type
BBB: Blood brain barrier
